# DNA Repair Protein HELQ and XAB2 as Chemoresponse and Prognosis Biomarkers in Ascites Tumor Cells of High-Grade Serous Ovarian Cancer

**DOI:** 10.1155/2022/7521934

**Published:** 2022-03-29

**Authors:** Fang Zhu, Siyu Yang, Ming Lei, Qiongqiong He, Lisha Wu, Yu Zhang

**Affiliations:** ^1^Department of Gynecology, Xiangya Hospital, Central South University, Changsha, Hunan, China; ^2^Gynecological Oncology Research and Engineering Center of Hunan Province, Changsha, Hunan, China; ^3^Department of Pathology, Xiangya Hospital, Central South University, Changsha, Hunan, China; ^4^Department of Pathology, School of Basic Medicine, Central South University, Changsha, Hunan, China; ^5^Institute of Medical Science, Xiangya Hospital, Central South University, Changsha, Hunan, China; ^6^National Clinical Research Center for Geriatric Disorders, Xiangya Hospital, Central South University, Changsha, Hunan, China

## Abstract

Nucleotide excision repair (NER) is an important mediator for responsiveness of platinum-based chemotherapy. Our study is aimed at investigating the NER-related genes expression in ascites tumor cells and its application in the prediction of chemoresponse in high-grade serous ovarian cancer (HGSC) patients. The relationship between 16 NER-related genes and the prognosis of ovarian cancer was analyzed in the TCGA database. NER-related genes including HELQ and XAB2 expressions were determined via immunocytochemistry in ascites cell samples from 92 ovarian cancer patients prior to primary cytoreduction surgery. Kaplan-Meier analysis and Cox model were used to investigate the association between NER-related gene expression and prognosis/chemotherapeutic response. Predicting models were constructed using a training cohort of 60 patients and validated in a validation cohort of 32 patients. We found that high expression of HELQ and XAB2 in the training cohort was associated with poor prognosis (for HELQ, *P* = 0.001, HR = 2.83, 95% CI: 1.46-5.49; for XAB2, *P* = 0.008, HR = 2.38, 95% CI: 1.23-4.63) and platinum resistance (for HELQ, *P* < 0.001; for XAB2, *P* = 0.006). In the validation cohort, the combination of HELQ and XAB2 (AUC = 0.863) showed the highest AUC. The expression levels of HELQ (RR 5.7, 95% CI 1.7-19.2) and XAB2 (RR 3.2, 95% CI 0.9-10.8) in ascites tumor cells were positively correlated to the risk of platinum resistance. In summary, we revealed that the expression levels of HELQ and XAB2 are candidate predictors for primary chemotherapy responsiveness and prognosis in HGSC. Ascites cytology is applicable as a promising method for chemosensitivity prediction in HGSC.

## 1. Introduction

Epithelial ovarian cancer (EOC) is the second most lethal gynecologic cancer worldwide [1], with a 5-year survival of 46%. Primary cytoreductive surgery followed by platinum-based chemotherapy has been the standard treatment of EOC over the past decades [2, 3]. However, chemoresistance is common in the later course of EOC. Unresponsiveness to chemotherapy is associated with poorer prognosis in EOC patients [4, 5]. Currently, the widely used predictor of the response to platinum-based chemotherapy in ovarian cancer has been the platinum-free interval (PFI). However, PFI is not a valid predictor. The PFI is a retrospective evaluation and may be influenced by the frequency and types of investigations a patient receives during follow-up. For platinum-resistant patients, they cannot benefit from treatment and have to endure the side effects of chemotherapy drugs. Therefore, the prediction of chemosensitiveness before primary treatment in EOC is a major clinical issue.

Platinum-based chemotherapy, such as carboplatin or cisplatin, causes DNA damage by intercalating DNA through interstrand cross-links (ICLs) between purine bases, resulting in DNA double-strand breaks (DSBs) [6, 7]. In response to genotoxic stress, cells activate the checkpoints to prevent further progression through the cell cycle and initiate DNA repair [8], whereas in cancer cells, inappropriate or aberrant activation of the DNA damage response network is associated with resistance to platinum [9, 10]. Previous studies have shown that NER was an important mediator for responsiveness of platinum-based chemotherapy. NER and high activity of NER was correlated with platinum resistance in EOC [11, 12]. Therefore, the identification of the key elements in NER pathways could provide biomarkers for early detection of platinum chemoresistance.

It is acknowledged that advanced stage EOC is prone to metastasize to the entire abdominal cavity via peritoneal dissemination and large amount of ascites generally ensues. Ascites cytology is a promising alternative to primary tumor tissue sampling, especially for the elderly or patients with poor general condition, in whom invasive procedures may be postponed due to comorbidities [13]. Zivadinovic et al. have observed good concordance between ascites cytology and primary tumor tissue sampling. The sensitivity of cytology was 98.92%, and the specificity was 93.6% [14]. It has been reported that the introduction of immunohistochemistry (IHC) staining of cell blocks obtained from ascitic fluid further improved that accuracy of diagnosis [15].

In this study, we first analyzed the correlation between NER-related genes and the prognosis of ovarian cancer cases from the TCGA database and found that the high-expression levels of Helicase POLQ-like (HELQ), Xeroderma pigmentosum group A-binding protein2 (XAB2), and replication protein A2 (RPA2) were associated with the poor prognosis of ovarian cancer. Then, we further evaluated the role of HELQ and XAB2 in ascites cell samples as a predictive biomarker. The predictive performance of ascites cytology was compared with paired primary tumor tissues.

## 2. Materials and Methods

### 2.1. Bioinformatics Analysis of TCGA Dataset

The normalized mRNA high-throughput sequencing data and clinical information of tubo-ovarian high-grade serous carcinoma were downloaded from The Cancer Genome Atlas (TCGA, https://portal.gdc.cancer.gov) via open access in December 2018. Sixteen NER-related genes (HELQ, ERCC1, ERCC3, ERCC4, ERCC5, ERCC6, ERCC8, DDB2, RAD23A, RAD23B, RPA1, RPA2, RPA3, XAB2, XPA, and XPC) were included in the study [16]. Patients who have not undergone platinum-based chemotherapy or incomplete follow-up information were excluded. The rest of 339 patients were divided into high and low according to the mRNA expression of each gene. The receiver operating characteristic (ROC) curve was employed to determine the optimal cut-off point for expression level. And the value at maximum Youden's index (sensitivity+specificity-1) was selected as the cut-off value [17, 18]. Survival analysis of overall survival (OS) and progression-free survival (PFS) was performed.

### 2.2. Study Cohorts and Clinical Information Collection

All eligible patients of the study were from the Xiangya Hospital of Central South University, China, between January 2014 to September 2019 and were treated in strict accordance with the version 1 2021 NCCN guidelines [19]. Eligible patients had to meet the following criteria: (1) The diagnosis of ovarian adenocarcinoma was reached by morphology and IHC of the ascites and tissue samples. The protocol for IHC staining was described in the study by Uehara et al. IHC stains that showed PAX8 (+), WT1 (+), CA125 (+), CK7 (+), CDX-2 (-), CK20 (-), and CEA (-) were recognized as ovarian origin [20], (2) treated by surgical debulking and histologically confirmed as high-grade serous ovarian cancer (HGSC), and (3) underwent at least three cycles of platinum-based chemotherapy after surgery. Patients who met the following criteria were excluded: (1) accompanied with other systemic malignancies; (2) received radiotherapy, chemotherapy, and biological therapy before cytological evaluation or debulking; (3) treated with platinum drugs other than cisplatin or carboplatin; (4) with incomplete clinical information; (5) loss of follow-up; and (6) lack of available biopsy specimens. A total of 92 patients were included in the study, including 60 in the training cohort and 32 in the validation cohort. Detailed description of the process of participants through the research was shown in Figure S1. The following clinical parameters were retrieved from medical records: age, pretreatment level of CA-125, volume of ascites, residual lesion, the International Federation of Gynecology and Obstetrics (FIGO) stage, chemotherapy regimen, OS, and PFS. Patient follow-up was terminated on November 1st, 2020. Patients were deemed as platinum resistant if they had disease progression during primary chemotherapy or disease recurrence within 6 months after completion of primary chemotherapy, while those without disease progression after 6 months from the end of primary chemotherapy were deemed as platinum sensitive. Surveillance was implemented upon completion of the initial treatment with 3-4 m interval during the first 2 years, 4-6 m interval from year 2 to year 3, 6 m internal from years 3-5, and annual visits after 5 years. These visits included symptom management, examination including a pelvic examination, chest/abdominal/pelvic CT and CA-125 or other tumor markers measurements. Subsequent imaging workup was indicated for patients with elevated CA-125, including ultrasound, CT, MRI, or PET-CT. If the lesion is found, it will indicate the recurrence or progression. PFI > 6 months predicts favorable response to retreatment; <6-month PFI is defined as platinum resistant [16, 21]. The study was approved by the Ethics Committee Xiangya Hospital of Central South University (No. 2017068222).

### 2.3. Ascites Cell Samples, Paired with Primary Tumor Tissues, Immunohistochemistry

To clarify the relationship between the expression levels of HELQ and XAB2 in ascites tumor cells and clinical characteristics in HGSC patients, ascites samples were obtained for immunocytochemistry staining. In newly diagnosed ovarian cancer patients, ascites was collected during peritoneal puncture before initial cytoreductive surgery. Firstly, all samples of ascites were submitted for routine cytologic examination. Approximately 20 to 50 ml ascites was spun down at 600 g for 5 minutes. After discarding the upper layer, the samples were fixed in 10% formalin overnight, embedded in paraffin and finally stained with hematoxylin-eosin (HE) or immunocytochemistry. All paired tumor tissue specimens were collected via surgical resection and paraffin-embedded for immunocytochemistry analysis in the Pathology Department of Xiangya Hospital. Paraffin-embedded ascitic fluid cells and tissue blocks were sliced into sections with a thickness of 2.5 *μ*m. Sections were dewaxed by turpentine, hydrated by gradient alcohol, and heated by microwave in citric acid buffer (pH = 6.0) at 100°C for 30 min to antigen retrieval. After natural cooling at room temperature, we used 3% hydrogen peroxide solution to block endogenous peroxidase and 5% bovine serum albumin to reduce nonspecific binding. After being washed once or twice in PBS, the sections were incubated with a HELQ antibody (Abclonal, A12661, 1 : 300) and a XAB2 antibody (Abcam, ab228006, 1 : 400) at 4°C overnight. The following day, the sections were washed twice in PBS and kept at room temperature for 1 h of secondary antibody incubation. The immunohistochemical reaction was observed with 3,3,0-diaminobenzidine (DAB), and hematoxylin was used for nuclear staining of all the tissue sections. Stained slides were scanned into digital images by the automatic scanning system. Five fields with highest positive expression were selected for each slice by 200x and 400x magnification and then analyzed by Vectra 2 system. All assessments were blinded with respect to clinical patient data.

### 2.4. Statistical Analysis

Kaplan-Meier analyses and log-rank test were used to analyze the OS and PFS of patients. Univariate analyses with enter method were performed by Cox regression survival analyses. The correlation between gene expression and clinicopathological features was estimated by the chi-square test, Fisher's exact test (for categorical variables), and binary logistic regression (for numerical variables). ROC curve analysis was used to assess the accuracy of the predicted probabilities. A *P* value of < 0.05 was considered statistically significant. All statistical analyses were performed with IBM-Microsoft SPSS version 22.0, GraphPad Prism 8.0, and R version 4.1.0.

## 3. Results

### 3.1. HELQ and XAB2 Were Associated with Poorer Prognosis of EOC Patients

**Figure 1:**
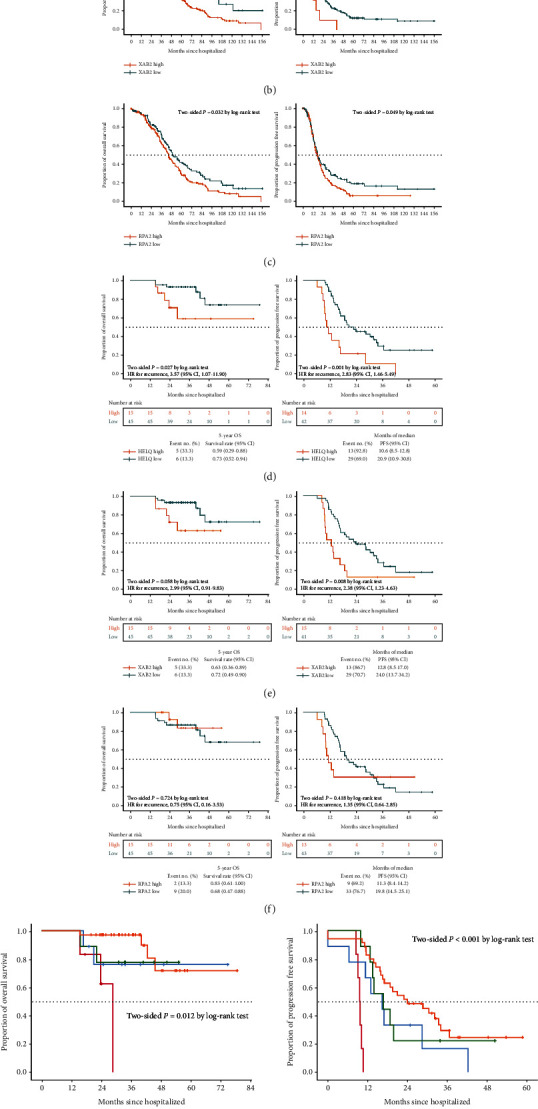
HELQ and XAB2 were associated with poor survival in patients with ovarian cancer. (a–c) Kaplan-Meier curves of OS and PFS according to the expressions of HELQ, XAB2, and RPA2 in TCGA tuboovarian high-grade serous carcinoma. (d–f) Kaplan-Meier survival curves for OS and PFS of patients with HGSC from the study cohort according to expressions of HELQ, XAB2, and RPA2 in ascites tumor cells. (g) Kaplan-Meier survival curves for OS and PFS of patients with HGSC from the study cohort according to HELQ-XAB2 stratification in ascites tumor cells. HELQ: helicase, POLQ like; RPA2: replication protein A2; XAB2: XPA binding protein 2; TCGA: The Cancer Genome Atlas; OS: overall survival; PFS: progression-free survival; HGSC: high-grade serous ovarian cancer.

We analyze the survival of 16 NER-related gene expressions in EOC patients from the TCGA database. The results showed that high expression levels of XAB2 (for OS, *P* = 0.049; for PFS, *P* = 0.012, Figure 1(b)) and RPA2 (for OS, *P* = 0.032; for PFS, *P* = 0.049, Figure 1(c)) were associated with poorer prognosis. According to our previous study demonstrating HELQ as a novel indicator of platinum-based chemoresistance for EOC [22], we included HELQ in this study, even though it was not statistically significant in this analysis (for OS, *P* = 0.312; for PFS, *P* = 0.334, Figure 1(a)). Details of 16 genes involved in the NER pathway are shown in Table S1. In the training cohort of 60 patients, better survival was strongly associated with low expression of HELQ (Figure 1(d)) and XAB2(Figure 1(e)) in ascites tumor cells. Given that RPA2 expression in ascites tumor cells (5-year survival rate, 0.83 vs 0.68, *P* = 0.724; median PFS, 11.3 months vs. 19.8 months, *P* = 0.418) was weakly correlated with poor prognosis of HGSC patients in training cohort (Figure 1(f)), it was not included in subsequent analyses.

Next, we stratified samples into 4 groups based on the combination of the HELQ and XAB2 expressions in ascites tumor cells: a high-expression HELQ/high-expression XAB2 group, a high-expression HELQ/low-expression XAB2 group, a low-expression HELQ/high-expression XAB2 group, and a low-expression HELQ/low-expression XAB2 group. We subsequently performed a survival analysis. Comparisons were made between the 4 groups. The median PFS was 22.6, 16.8, 16.6, and 15.0 months, respectively (*P* < 0.001), and the median OS was 55.1, 49.5, 38.6, and 38.6 months (*P* = 0.012), respectively (Figure 1(g)).

### 3.2. High Expression of HELQ and XAB2 in Ascites Tumor Cells Were Correlated with Platinum Resistance in HGSC Patients

The characteristics of the 60 HGSC patients were summarized in Table 1. Subsequent analyses of HELQ and XAB2 expressions in ascites tumor cells and clinical data showed statistically significant increased distribution of platinum resistance in patients with high-expression of HELQ (*P* < 0.001) and XAB2 (*P* = 0.006). Other clinical features such as age, stage, and residual disease did not harbor any significant distribution variation.

**Figure 2:**
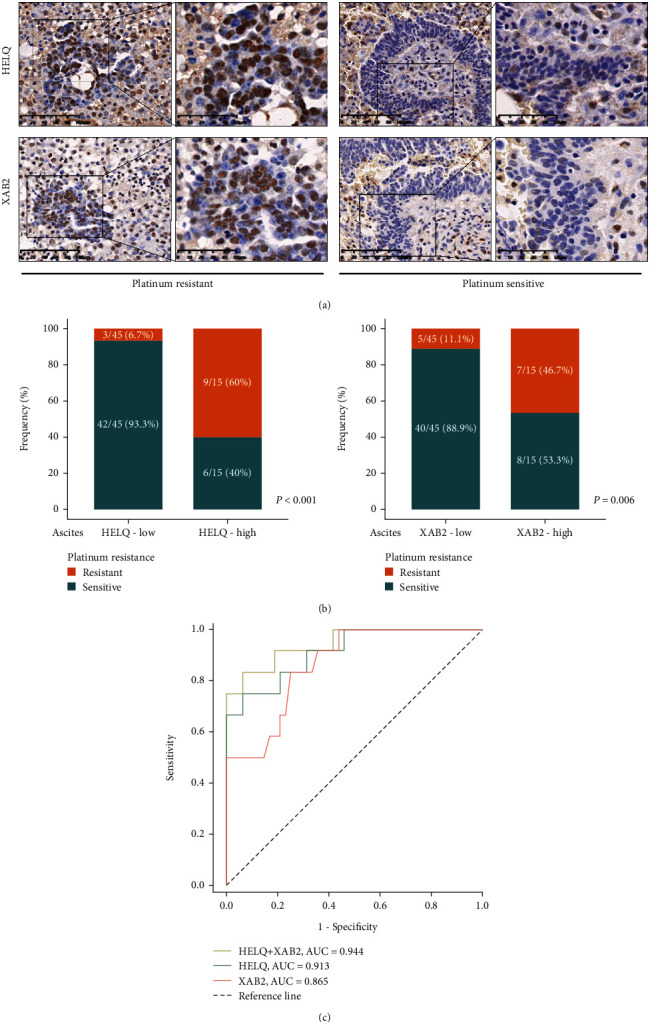
HELQ and XAB2 expressions in ascites tumor cells associated with platinum-resistant phenotype. (a) Representative immunohistochemistry images of HELQ and XAB2 in patients with platinum-resistant and platinum-sensitive phenotypes. (b) Frequency of platinum-resistant patients from the study cohort was compared according to HELQ and XAB2 expressions. (c) Receiver operator characteristic curves with AUC according to relative expressions of HELQ and XAB2 in ascites tumor cells of patients with HGSC. AUC: area under the curve.

To confirm the correlation between HELQ and XAB2 expressions and platinum-based chemotherapy response in HGSC, we noticed that high expression of HELQ and XAB2 in ascites tumor cells were strongly correlated with platinum resistance (Figure 2(a)). In addition, we compared the frequency of platinum-resistant phenotype in cases with high or low expression level of HELQ and XAB2. We observed platinum resistance enrichment in the high expression of HELQ (9/15 vs. 3/45, *P* < 0.001) and XAB2 (7/15 vs. 5/45, *P* = 0.006) (Figure 2(b)), suggesting that HELQ and XAB2 expressions in ascites tumor cells could be predictors of platinum resistance in HGSC.

Then, we investigated the relative expression of HELQ and XAB2 in ascites tumor cells utilizing ROC curves, to evaluate the performance of HELQ and XAB2 as predictors. ROC curves for HELQ alone, XAB2 alone, and combination of HELQ and XAB2 demonstrated the highest area under the curve (AUC) for the combination of HELQ and XAB2 (AUC = 0.944), followed by HELQ alone (AUC = 0.913) and lastly by XAB2 alone (AUC = 0.865) (Figure 2(c)).

Table 2 showed the diagnostic performance of HELQ and XAB2 expression levels in ovarian cancer with platinum resistance in study cohort. Positive predictive values (PPV) for high expression of HELQ and XAB2 individually in platinum resistance were 60% and 46.7%, respectively. PPV for platinum resistance improved (100%) when using dual markers.

### 3.3. Expression of HELQ and XAB2 in Ascites Tumor Cells Was Positively Correlated with Chemoresistance in HGSC Patients in Validation Cohort

**Figure 3:**
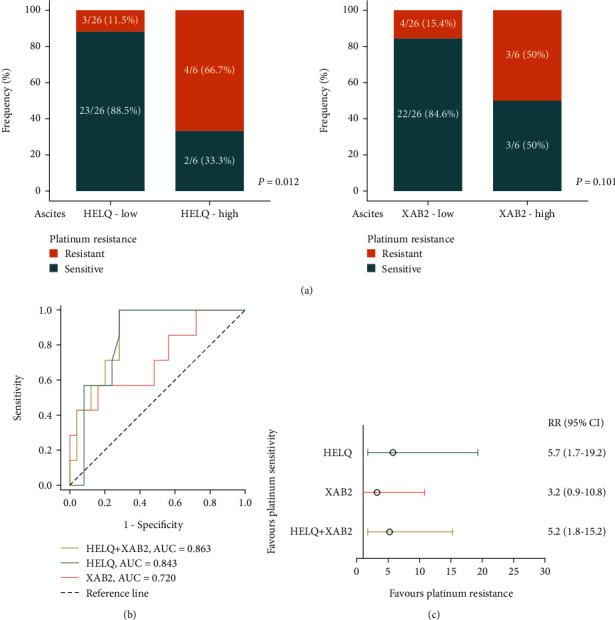
Expressions of HELQ and XAB2 in ascites tumor cells were positively correlated with chemoresistance of HGSC patients in validation cohort. (a) Frequency of platinum-resistant patients from the validation cohort was compared according to HELQ and XAB2 expressions. (b) Receiver operator characteristic curves with AUC according to relative expressions of HELQ and XAB2 in ascites tumor cells of patients with HGSC. (c) Forest plot for relative risk of the high expressions of HELQ and XAB2 in ascites tumor cells. RR: relative risk.

The clinicopathologic characteristics of 32 HGSC in the validation cohort were summarized in Table 3. A higher frequency of platinum resistance in patients with high expression of HELQ (4/6 vs 3/26, *P* = 0.012) was observed in validation cohort. However, the platinum resistance enrichment in the high expression of XAB2 was insignificant (3/6 vs. 4/26, *P* = 0.101) (Figure 3(a)). Consistent with the study cohort, ROC curves showed the highest AUC for the combination of HELQ and XAB2 (AUC = 0.863), followed by HELQ alone (AUC = 0.843) and lastly by XAB2 alone (AUC = 0.720) (Figure 3(b)). We observed a 5.7 times higher risk of developing platinum resistance in cases with high expression of HELQ in ascites tumor cells (relative risk (RR) 5.7, 95% CI 1.7-19.2). The platinum resistance risk was also higher in cases with high expression of XAB2 (RR 3.2, 95% CI 0.9-10.8) and with coexpression of HELQ and XAB2 (RR 5.2, 95% CI 1.8-15.2) (Figure 3(c)).

**Figure 4:**
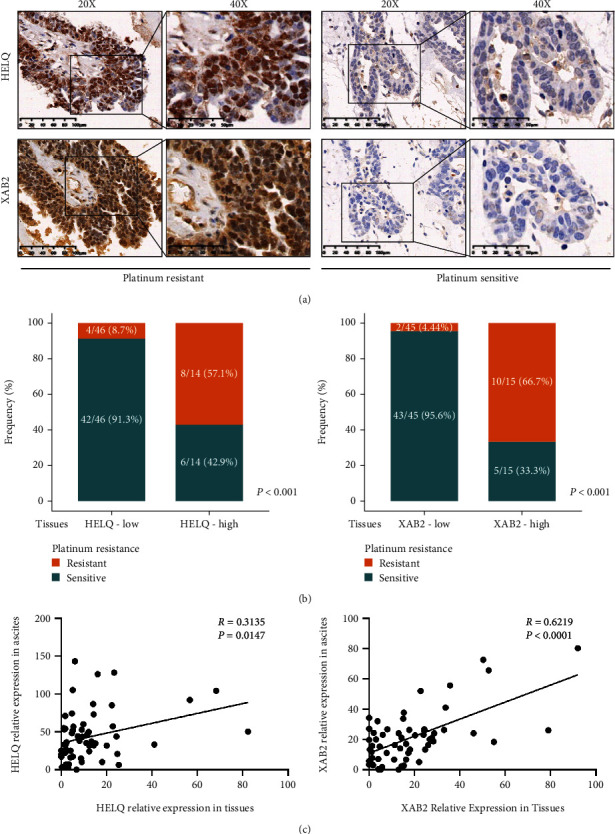
HELQ and XAB2 expressions in tumor tissues were correlated with that in ascites tumor cells. (a) Representative immunohistochemistry images of HELQ and XAB2 in tumor tissues of HGSC patients with platinum-resistant and platinum-sensitive phenotypes. (b) Frequency of platinum-resistant patients from the study cohort was compared according to HELQ and XAB2 expressions in tumor tissues. (c). Expressions of HELQ and XAB2 in ascites tumor cells were used to analyze the correlation with that in matched tumor tissues.

To validate the reliability of HELQ and XAB2 results in ascites tumor cells, we also determined the expression of HELQ and XAB2 in paired primary tumor tissues (Figure 4(a)). Consistent with ascites samples, a trend toward platinum resistance enrichment was observed in cases with high expression of HELQ and XAB2 in paired tumor tissues (Figures 4(b) and 4(c)), which supported HELQ and XAB2 as predictors of platinum resistance.

## 4. Discussion

Platinum-based chemotherapy has drastically improved the prognosis of EOC patients. Unfortunately, resistance to platinum drugs frequently occurs and limits the efficacy of chemotherapy. However, the absence of an effective predictor of chemoresistance prior to systemic therapy initiation has resulted in patients receiving unindividualized chemotherapy regimen regardless [23, 24]. DNA damage repair (DDR) plays a critical role in the occurrence and development of numerous cancers [25–28]. Abnormal activation of DNA damage repair, such as the NER pathway, has been confirmed to be associated with the prognosis and platinum resistance in ovarian cancer [22, 29]. Previous study had confirmed that the high expression of HELQ in EOC tissues was associated with poor prognosis and platinum resistance [30].

70% of ovarian cancer is diagnosed at advanced stages, accompanied by extensive pelvic-abdominal metastasis and large amounts of ascites [31, 32]. Systemic therapy is indicated for these patients, and it is of great importance to obtain an accurate pathological diagnosis prior to the initiation of treatment. Ascites cytology, as a less invasive and more accessible alternative to primary tumor tissue biopsy, also provides prediction of chemoresistance. Therefore, the number of studies concerning ascites in ovarian cancer has been increasing. Goto et al. suggested that p16INK4a expression in ascites cells was a candidate marker in predicting primary response to chemotherapy and prognosis [4]. However, concerns have been raised about the application of ascites cytology in ovarian cancer regarding its reliability [33]. Previously, ascites cytology has been investigated as part of the diagnostic module for ovarian cancer and its efficacy has been proved [34, 35]. Additionally, ascites cytology has demonstrated noninferiority to primary tumor tissue sampling and blood samples in the determination of the patient's BRCA status [36, 37]. Our current finding showed that HELQ and XAB2 expressions in the ascites tumor cells correlated with HGSC patient's response to platinum-based chemotherapy and clinical outcomes indicate the potentiality of HELQ and XAB2 as independent biomarkers to predict HGSC patients' response to platinum drugs. More importantly, we observed good accordance of HELQ and XAB2 expressions between ascites tumor cells and paired tumor tissues tumor tissues in our study. Hence, the assessment of HELQ and XAB2 expression levels in ascites tumor cells may help clinicians to design individualized treatment strategies for HGSC patients.

Our study has confirmed that ovarian cancer patients with the high expression of HELQ or XAB2 had decreased PFS and OS, respectively. HELQ, an ATP-dependent 3′-5′ DNA helicase, plays a pivotal role in DNA processing, including homologous recombination (HR) repair [30], by regulating related proteins in the NER pathway which, in turn, contributes to cellular response to cisplatin and patients' response to platinum-based chemotherapy [20]. In this study, the high expression of HELQ and XAB2 in ascites tumor cells may lead to an increase of the ability of DNA damage repair, such as the HR or NER pathway, and a decrease in apoptosis, which led to tumor cell tolerance to platinum drugs. As a member of the NER pathway, XAB2 protein participates in many biological processes such as transcription-couple DNA repair, ATRA-induced cellular differentiation, splicing, mRNA export, and transcription [38, 39]. Recent studies indicated that XAB2 also participated in the end step of HR [40]. However, the mechanism of HELQ and XAB2 leading to platinum resistance in ovarian cancer needs further exploration.

This study was limited by a relatively small number of cases and possible selection bias. Further analysis by a large prospective study is needed to confirm our findings. However, the results of our study suggested the assessment of HELQ and XAB2 expression levels in cytology of ascites could be a less invasive and convenient predictive method in HGSC especially in consideration of chemotherapy.

## 5. Conclusion

In summary, our findings demonstrated that immunocytochemistry for HELQ and XAB2 expressions in ascites tumor cells are applicable in prediction of the primary response to chemotherapy and prognosis. We recommend a large multicenter prospective study to confirm the clinical significance of HELQ and XAB2 in ascites tumor cells in HGSC be performed.

## Figures and Tables

**Table 1:** Clinicopathologic characteristics of 60 HGSC patients in Xiangya hospital.

Clinicopathologic parameters	Frequency (%)	Expression level in ascites tumor cells					
		HELQ			XAB2		
		High	Low	*P*	High	Low	*P*
Age (year)				0.153			>0.99
≤60	48 (80)	10	38		12	36	
>60	12 (20)	5	7		3	9	
FIGO stage				—			—
I-II	3 (5)	0	3		0	3	
III-IV	57 (95)	15	42		15	42	
Residual disease				0.637			0.428
R0	14 (23)	2	12		2	12	
R1	29 (48)	8	21		7	22	
>R1	17 (28)	5	12		6	11	
Ascitic fluid (ml)				0.313			0.313
≤500	15 (25)	2	13		2	13	
>500	45 (75)	13	32		13	32	
Chemotherapy response				<0.001			0.006
Sensitive	48 (80)	6	42		8	40	
Resistant	12 (20)	9	3		7	5	

*P* value was calculated by chi-square test. HGSC: high-grade serous ovarian cancer; FIGO: International Federation of Gynecology and Obstetrics. Statistically significant (*P* < 0.05).

**Table 2:** Diagnostic performances of HELQ and XAB2 expression levels in ovarian cancer with platinum resistance.

Triage	Diagnostic accuracy (95% CI)			
	Sensitivity	Specificity	PPV	NPV
High expression of HELQ	75% (43-93)	87.5% (74-95)	60% (33-83)	93.3% (81-98)
High expression of XAB2	58.3% (29-84)	83.3% (69-92)	46.7% (22-73)	88.9% (75-96)
High expression of both HELQ and XAB2	50% (22-78)	100% (91-100)	100% (52-100)	88.9% (77-95)

PPV: positive predictive value; NPV: negative predictive value.

**Table 3:** Clinicopathologic characteristics of 32 HGSC patients in validation cohort.

Clinicopathologic parameters	Frequency (%)	Expression level in ascites tumor cells					
		HELQ			XAB2		
		High	Low	*P*	High	Low	*P*
Age (year)				0.590			0.590
≤60	25 (78)	4	21		4	21	
>60	7 (22)	2	5		2	5	
FIGO stage				1.00			1.00
I-II	1 (4)	0	1		0	1	
III-IV	31 (97)	6	25		6	25	
Residual disease^∗^				0.049			0.040
R0	13 (41)	0	13		0	13	
R1	12 (38)	4	8		4	8	
>R1	6 (19)	1	5		2	4	
Ascitic fluid (ml)				0.361			0.059
≤500	13 (41)	1	12		0	13	
>500	19 (59)	5	14		6	13	
Chemotherapy response				0.012			0.101
Sensitive	25 (78)	2	23		3	22	
Resistant	7 (22)	4	3		3	4	

^∗^With one patient whose residual disease was unavailable. HGSC: high-grade serous ovarian cancer; FIGO: International Federation of Gynecology and Obstetrics.

## Data Availability

Publicly available datasets were analyzed in this study (https://portal.gdc.cancer.gov).

## Supplementary Materials

Supplementary Materials Figure S1. the flow diagram of high-grade serous ovarian cancer patients. Table S1: correlation between NER gene expression and survival in 339 ovarian cancer patients from TCGA.
