# Supplementary material for: DNA Repair Protein HELQ and XAB2 as Chemoresponse and Prognosis Biomarkers in Ascites Tumor Cells of High-Grade Serous Ovarian Cancer
Source: J Oncol. 2022 Mar 29;2022:7521934. doi: 10.1155/2022/7521934 (PMC8983184; doi:10.1155/2022/7521934)

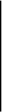

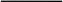

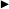

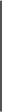

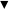

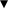

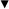

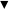

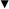


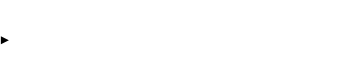


| Excluded  ♦ Lost to follow up (n=5)  ♦ Evaluation time not reached （n=5） |
| --- |


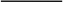


| Potentially eligible patients (n=416) |
| --- |

| Excluded  ♦ Non-ovarian cancer (n=172)  ♦ Lack of ascites cell samples （n=135）  ♦ Less than 3 times of chemotherapy （n=7） |
| --- |

| Patients joined in follow-up (n=102) |
| --- |

| 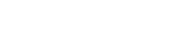 Eligible patients (n=92) |
| --- |

| Clinical responses were measured  according to PFI interval. |
| --- |

| 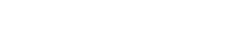 Platinum-Resistance (n=19) |  | 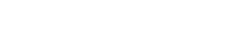 Platinum- Sensitive (n=73) |
| --- | --- | --- |

**Figure** **S1.** **The** **flow** **diagram** **of** **high-grade** **serous** **ovarian** **cancer** **patients.**


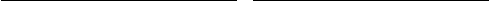

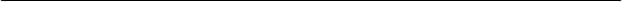

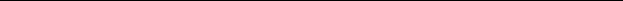


**Table** **S1 Correlation** **between** **NER** **genes** **expression** **and** **survival** **in** **339** **ovarian** **cancer** **patients** **from** **TCGA.**


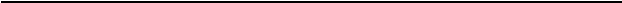


**PFS** **(months)** **OS** **(months)**

**Gene** **N** **Median** **95%** **CI** ***P*** **Median** **95%CI** ***P***

**ERCC1**

High

Low

**ERCC2**

High

Low

**ERCC3**

High

Low

**ERCC4**

High

Low

**ERCC5**

High

Low

**ERCC6**

High

Low

**ERCC8**

High

Low

**DDB2**

High

Low

**RAD32A**

High

Low

**RAD32B**

High

Low

**RPA1**

High

Low

**RPA2**

High

Low

80

259

3

336

41

298

235

104

20

319

179

160

5

334

18

321

83

256

26

313

6

333

206

133

18.4

18.2

9.1

18.3

20.4

18.1

17.8

19.3

22.3

18.2

18.4

17.1

9.1

18.2

16.0

18.3

19.2

18.1

15.6

18.3

14.9

18.2

17.9

18.2

0.558

15.7-21.1

15.3-21.2

0.006**

0.6- 17.7

16.1-20.4

0.601

16.7-24.2

16.1-20.2

0.529

15.7-20.0

15.5-23.0

0.848

10.8-33.7

16.5-20.0

0.613

16.33-20.5

14.5- 19.8

0.116

0.6- 17.7

16.2-20.3

0.082

14.0- 18.0

16.0-20.6

0.576

16.3-22.1

15.7-20.5

0.268

13.5- 17.8

16.0-20.6

0.121

8.2-21.7

15.9-20.6

0.049*

15.4-20.4

15.2-21.2

46.1

48.2

45.1

50.4

45.6

83.0

-

46.3

45.3

48.1

44.1

48.3

45.5

49.5

48.2

45.6

45.6

57.5

49.0

46.0

49.0

45.5

45.1

50.0

0.239

42.3-50.0

41.5-54.9

0.043*

40.2-50.1

43.4-57.3

0.206

42.3-49.0

34.7- 131.3

0.021*

-

42.9-49.8

0.096

34.9-55.7

44.4-51.7

0.003**

31.8-56.5

43.5-53.1

0.094

41.0-50.0

29.4-69.6

0.760

41.9-54.6

39.9-51.4

0.075

41.9-49.3

24.2-90.8

0.448

41.5-56.5

42.2-49.8

0.664

43.5-54.5

41.7-49.4

0.032*

40.6-49.7

40.0-59.9

**Table** **S1:** **Correlation** **between** **NER** **genes** **expression** **and** **survival** **in** **339** **ovarian** **cancer** **patients** **from** **TCGA** **(continued).**


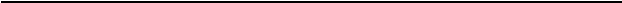


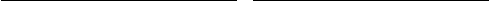
**PFS** **(months)** **OS** **(months)**

**Gene** **N** **Median** **95%** **CI** ***P*** **Median** **95%CI** ***P***


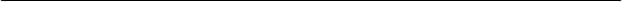


**RPA3**

High

Low

**XAB2**

High

Low

**XPA**

High

Low

**XPC**

High

Low

72

267

14

325

104

235

21

318

18.3

18.1

12.5

18.3

18.4

18.1

19.4

18.1

14.5-22.1 15.9-20.4

9.0- 16.0 16.2-20.4

15.8-21.0 15.7-20.6

16..7-22.2

16.1-20.1

0.583

45.3

50.4

0.012*

45.5

57.5

0.622

46.3 45.5

0.767

50.5

45.6

0.626

41.1-49.5

40.0-60.8

0.049*

41.7-49.2

34.9-80.1

0.397

42.83-49.8

25.0-66.0

0.333

34.4-66.7

42.1-49.2


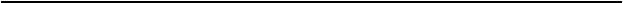

Supplement: Supplementary Materials — Figure S1. the flow diagram of high-grade serous ovarian cancer patients. Table S1: correlation between NER gene expression and survival in 339 ovarian cancer patients from TCGA. [file 7521934.f1.docx]
